# Demobilisation and the Y.M.C.A.

**Published:** 1919-06-28

**Authors:** 


### DEMOBILISATION AND THE Y.M.C.A.

### By A SOCIAL WORKER.

"Lest we forget" what a good gift of God's hand is this amazing summer-in-spring, which has brought us the joy and the healing influences of warm air and sun before we could dare expect them, let us remember the weather of a month ago. On April 28 the present writer drove out from the city of Lille, through the once busy industrial towns that are now rubbish-heaps, in a dizzying snowfall. The hedges were green, and on them snow lay like a fulness of their own blossom, and its merciful whiteness was trying to cover up the ghastly mounds that once were homes. In many places a village was so effaced and already so grass-grown that only a large board painted with its name told travellers where they were. In the coal-mining town of Lens gaunt shafts of grey stone marked the church's site; the rest was a kind of red gravel, where once brick houses had stood, and beyond were seen the broken pit-head shafts of the flooded mines. Over the meadows and the livid stumps that marked where in other springs had been blossoming orchards, rusty barbed wire was everywhere tangled, and water-filled shell-holes added a look of some hideous disease affecting the land. But everywhere over the vast area men were at work in the pitiless chill under the leaden sky that seemed in such complete accord with the world's misery.

### The Battle of Peace.

Men of all nations—our own, Frenchmen, and gangs of emerald-clad, sullen-faced German prisoners, Chinese and Japanese, Malagasy and Algerians, Swedes, Portuguese, Americans of course, all fighting that battle of peace which meant the overcoming of ruin and desolation, and "reconstruction" of a habitable world.

Yes, but what of those who are working in that atmosphere of desolation? Working, too, at the peril of their lives, for the whole country is strewn with unexploded shells, hand grenades packed with melinite, and other dangerous toys. The very soil—*Si on le remue ici et là, ça pue*, said a Frenchman, one who stood some way off read the words in the sickened look on his face rather than heard them. When work is done still the sight of human misery fills the picture, the *refugiés* who come back are prowling round and over the heaps—and that, too, is dangerous business—to find the bit of rubbish that represents home, these are the only inhabitants to talk with. Dusk falls, and darkness mercifully shuts out the desolation which morning will show again. Tommy, with his wonderful cheeriness, will perhaps go the length of telling you he is "a bit fed up" when you see him there, when he comes home he will tell his mother he "was a bit fed up." He will not tell, because he cannot—and if he could he would not want the home folks to know what terrible depths of depression are camouflaged by that light-hearted phrase. But those who have seen his circumstances for themselves have seen also what a hut means to him. At midday on April 28 the snow was melting into a cold pea-soup on the road, where it mingled with that indescribable thing for which no adjectives really exist—the mud of Flanders. But at cross-roads in the wreck of yet another town there was a hut, and the men's faces as they sat in it and wrote the countless letters Tommy does write, or read papers or drank steaming soup or cocoa, had a look of *détente* which spoke volumes. You need perhaps to go abroad, to France or to Mesopotamia or Archangel, really to appreciate the value of the Red Triangle over a building. But you may have at least a first easy lesson in London if you realise that in one recent week 44,486 men slept in the huts there, and if imagination, aided by a little knowledge of London's underworld, can enable you to picture what it is to be there alone, young, unemployed for the moment, and a stranger.

It seems a pity to have to refer to some recent allegations and reflections upon the work of the Y.M.C.A. Such things happen, and the Society no doubt took the wisest course in inviting a committee of three gentlemen to look into the whole matter and report. It is probably good that public attention should be drawn to the fact that in England 10.2 per cent., and in France 7.9 per cent. on the turnover represented the whole expenditure on the salaries and keep of workers. It was no doubt desirable also that the whole scheme for education in the Army, with the exception of popular lectures and such matters as music teaching and informal study classes should pass into Government hands. According to our unfailing British custom, a voluntary society blazed the trail, and the road was made and worked by official authority. But the period of demobilisation continues to be a very anxious one. The soldier has to be turned into a civilian, and it is of infinite importance to the nation that he should be a better citizen than he was before. Otherwise, may he not risk becoming a much worse one? If, therefore, the Y.M.C.A. makes now a final appeal for funds, it is not because workers are unwilling to resign a now effete work to which they have become attached, but because there was never more pressing need of it.

### Its Widespread Activity.

At the request of the military authority, for instance, the Association has now a worker and what is practically a Y.M.C.A. on every boat which goes down the Rhine carrying men to be demobilised, 600 a day at present, while the hall commandeered at Cologne, which holds 2,000, is crowded every night, and classes and lectures go on in a smaller hall. And the Army of Occupation has sixty-eight such centres, large and small. Belgium, too, is dotted with them. France has 240 centres; athletics are here proving their value; 127 different plays have been produced by the men; music is a ceaseless joy and uplift, and the ladies of the Red Triangle find a ready response to their effort to teach old country dances. At the military hospitals in France, the Y.M.C.A. hostel offers hospitality to the families of men dangerously ill.

In Russia, "it is not too much to say," the recent Bulletin notes, "that but for the fifty centres, the depression among our troops, which became manifest from the time of signing the Armistice, would have assumed a much more serious aspect." We can think what the visit of a Y.M. worker, bringing books, comforts, news, must be to groups of men on lonely outposts in the forests and on the shores of the White Sea. And so in Italy, in the Balkans, Mesopotamia, India, where a sweeper lately sent back word from Mesopotamia that his new-born grandson should be named Y.M.C.A.!

For the men of other nations, too, benefit by the Red Triangle. On that same dreary April 28 Chinese labourers —on the ground that rises up and up past Plœgsteert till it comes to the silent horror of Ypres—Chinese labourers went in and out under the quaint wooden arch, with its inscriptions, that leads to their camp.

No; the Y.M.C.A. has not finished its war work; it needs our sympathy, practical help, and prayers, that it may carry on through demobilisation and make of the most of those who are helped helpers in their turn for peace-time needs.

